# Association of Human Bocavirus 1 Infection with Respiratory Disease in Childhood Follow-up Study, Finland

**DOI:** 10.3201/eid1802.111293

**Published:** 2012-02

**Authors:** Mira Meriluoto, Lea Hedman, Laura Tanner, Ville Simell, Marjaana Mäkinen, Satu Simell, Juha Mykkänen, Jan Korpelainen, Olli Ruuskanen, Jorma Ilonen, Mikael Knip, Olli Simell, Klaus Hedman, Maria Söderlund-Venermo

**Affiliations:** University of Helsinki, Helsinki, Finland (M. Meriluoto, L. Hedman, M. Knip, K. Hedman, M. Söderlund-Venermo);; Haartman Institute, Helsinki (M. Meriluoto, L. Hedman, K. Hedman, M. Söderlund-Venermo);; Helsinki University Central Hospital, Helsinki (L. Hedman, M. Knip, K. Hedman);; University of Turku, Turku, Finland (L. Tanner, V. Simell, M. Mäkinen, S. Simell, J. Mykkänen, J. Korpelainen, O. Ruuskanen, J. Ilonen, O. Simell);; University of Eastern Finland, Kuopio, Finland (J. Ilonen);; Tampere University Hospital, Tampere, Finland (M. Knip);; Folkhälsan Research Center, Helsinki (M. Knip)

**Keywords:** respiratory illness, disease association, serology, virus infection, etiology, parvovirus, bocavirus, viruses, Finland

## Abstract

Human bocavirus 1 (HBoV1) DNA is frequently detected in the upper airways of young children with respiratory symptoms. Because of its persistence and frequent co-detection with other viruses, however, its etiologic role has remained controversial. During 2009–2011, using HBoV1 IgM, IgG, and IgG-avidity enzyme immunoassays and quantitative PCR, we examined 1,952 serum samples collected consecutively at 3- to 6-month intervals from 109 constitutionally healthy children from infancy to early adolescence. Primary HBoV1 infection, as indicated by seroconversion, appeared in 102 (94%) of 109 children at a mean age of 2.3 years; the remaining 7 children were IgG antibody positive from birth. Subsequent secondary infections or IgG antibody increases were evident in 38 children and IgG reversions in 10. Comparison of the seroconversion interval with the next sampling interval for clinical events indicated that HBoV1 primary infection, but not secondary immune response, was significantly associated with acute otitis media and respiratory illness.

## Abstract 2

Since its discovery in 2005, human bocavirus type 1 has often been found in the upper airways of young children with respiratory disease. But is this virus the cause of the respiratory disease or just an innocent bystander? A unique study in Finland, which examined follow-up blood samples of 109 healthy children with no underlying illness starting at birth and until they were 13 years of age, found that acute bocavirus infection resulted in respiratory disease. All children had been infected by age 6. Most retained their antibodies to this virus; some lost them. Children who were later re-exposed to bocavirus did not get sick from this virus. Thus, human bocavirus type 1 is a major cause of respiratory disease in childhood.

Human bocavirus 1 (HBoV1), a new member of the *Bocavirus* genus of the family *Parvoviridae*, was discovered in 2005 by large-scale sequencing in nasopharyngeal samples from children (*1*). HBoV1 DNA has since been frequently detected by PCR in the upper airways of young children who have upper or lower respiratory tract illness (URTI, LRTI) and, less frequently, in their feces (*2**,**3*). Furthermore, 3 other bocaviruses, HBoV2, 3, and 4, were recently detected in human feces (*4**–**6*), and HBoV2 has been associated with acute gastroenteritis (*5*).

HBoV1 in the upper airways also occurs persistently or recurrently in asymptomatic children (*7**–**11*). Because of these characteristics and frequent co-detection with other viruses, the role of HBoV1 in respiratory illness has been questioned. Circumventing the PCR-related problems of prolonged or recurrent positivity and disclosing the association of HBoV1 infection with disease require a more reliable diagnosis that uses serum for PCR and antibody detection (*12**–**16*). By using serology, one can distinguish between primary and secondary HBoV1 infections. We recently detected secondary HBoV1 immunoactivations in immunocompetent adults (*17*), but no data exist on the clinical effects of such events or on their frequency in children. Furthermore, to our knowledge, no prospective studies with reliable diagnostics have been conducted to determine the clinical associations of primary HBoV1 infection.

We determined HBoV1 primary infection in relation to clinical symptoms among constitutionally healthy children who were serologically followed from infancy up to age 13 years. In addition, we investigated the kinetics of HBoV1 viremia and IgG and IgM antibody responses, IgG avidity maturation, and the occurrence and clinical effects of secondary infections or immunoactivations.

## Materials and Methods

### Patients and Samples

We conducted this study during 2009–2011. Participants were from the ongoing population-based Diabetes Prediction and Prevention (DIPP) study, a prospective survey of the preclinical events preceding type 1 diabetes among genetically susceptible children in Finland (*18**,**19*). These children, who carry specific human leukocyte antigen (HLA)–DQ genotypes conferring increased susceptibility to type 1 diabetes, were observed from birth for the appearance of diabetes-associated antibodies and viral infections. By the end of 2002, a total of 68,953 newborn children (27,030 in Turku) had been tested for their HLA-conferred risk for type 1 diabetes. From this group, 10,743 (4,391 in Turku) were invited to join the DIPP study, and 8,014 (2,942) of these participated.

The 109 DIPP children in this study were randomly chosen (computer algorithm that gives equal relative amounts of all HLA types studied) from children born during 1995–2002 in Turku, fulfilling the following criteria: 1) to ensure that all their samples were not contaminated or otherwise compromised (e.g., multiple thaws), these children's samples had never been used in any previous studies; 2) participating children had been followed up according to the sampling schedule as promptly as possible; and 3) the children had to be of normal health and did not have type 1 diabetes or any diabetes-related antibodies by the end of 2002. Of these 109 constitutionally healthy children, 56 were girls. We analyzed the children’s 1,952 serum samples (mean of 18 samples per child, median 17, range 12–27), obtained from the average age of 3 months (median 0.31 years, range 0.20–0.91 years) to an average of 8 years (median 8.5 years, range 4–13 years), as well as umbilical cord blood samples from 9 selected children. The 109 children were examined at a mean interval of 110 days (median 96 days, range 55–484 days) until age 2 years and subsequently at a mean interval of 197 days (median 182 days, range 92–849 days) until October 2008 (unless they were discontinued earlier). At each examination, a serum sample was drawn, divided into aliquots, and stored at –70°C. All serum samples were tested for IgG and IgM antibodies against HBoV1. HBoV1 IgG avidity and HBoV1 quantitative PCR (qPCR) were conducted on the 3 specimens flanking each serodiagnosis (primary and secondary IgG antibody increases). At each child’s scheduled visit, the parents completed a questionnaire and were interviewed by a study nurse about any clinical symptoms or illnesses since the previous visit. Acute otitis media (AOM), sinusitis, tonsillitis and LRTI were diagnosed by a physician. All prescribed antimicrobial drugs were also recorded. Thirty (28%) children had physician-diagnosed allergic diseases, e.g., asthma; diabetes-associated autoantibodies developed in 7 (6%) children, but none of these children progressed to clinical diabetes.

The ethics committee of the Hospital District of Southwest Finland approved the study protocol. The legal guardians of the study participants provided written informed consent.

### Enzyme Immunoassays

HBoV1 IgG and µ-capture IgM enzyme immunoassays (EIAs) were conducted as described (*13*) with biotinylated virus–like viral protein 2 particles as the antigen. The diagnostic sensitivity was 97% and specificity 99.5% for these EIAs done in combination (*13*). Our diagnostic criteria for HBoV1 primary infection were seroconversion or PCR positivity in serum occurring for the first time; and for an HBoV1 secondary immunoactivation, a >4-fold titer increase in IgG antibodies in 2 adjacent serum samples after seropositivity. We used a protein-denaturing EIA to analyze HBoV1 IgG avidity (*17*). All samples from each child were studied in parallel.

### Real-time qPCR

The DNA in 20 µL of serum was extracted by phenol-chloroform, precipitated by sodium acetate and ethanol, and then eluted in 20 µL of 10 mmol/L Tris-Cl buffer (pH 8.0); 5 µL was assayed by using PCR. The HBoV1 nucleoprotein 1 (NP1) gene-based qPCR was performed as described (*12**,**20*) with a Stratagene Mx3005P instrument (Agilent Technologies, Santa Clara, CA, USA). The quantification standard was a plasmid (pSt2; GenBank accession no. DQ000496) containing the HBoV1 NP1 gene comprising serial dilutions covering 7 logs. Water served as negative controls and pSt2 as the positive control. The 3 serum samples from each child, including and flanking the seroconversion or secondary increase(s), were studied in parallel.

### Statistics of Clinical Correlates

Any infection-related illnesses—URTI (with fever or >2 respiratory symptoms), LRTI, fever without respiratory tract infection, tonsillitis, AOM, conjunctivitis, sinusitis, gastroenteritis (with vomiting or diarrhea), exanthema with fever, and other infection-related illnesses—during the HBoV1 primary infection or during the secondary immune response were compared with illnesses during the previous sample interval and the subsequent interval in each child. One child, for whom complete clinical information was lacking, was excluded. Liddell exact test served for statistical analyses, and p values <0.05 were considered significant.

Additionally, we compared the stability of or decrease in IgG antibodies after conversion in each child during the entire study, with the presence of allergic diseases, diabetes-associated antibodies, symptoms at HBoV1 primary infection, and with the child’s increased susceptibility to infections in general, as defined by the pediatric infectious disease specialists of Turku University Hospital on the basis of clinical features in primary immunodeficiency diseases (*21*), by the number of infection episodes (>10 AOM, >2 acute sinusitis, >1 pneumonia or >1 acute pyelonephritis), tonsillectomy or insertion of tympanostomy tubes because of recurrent infections. We used the Fisher exact test and SAS version 9.2 (SAS Institute, Cary, NC, USA) for these statistical analyses.

## Results

### HBoV1 Immune Response

**Figure 1:**
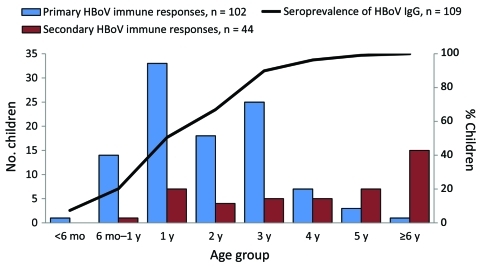
Age distribution of children with primary and secondary human bocavirus (HBoV) immune responses and seroprevalence of HBoV1 IgG, Finland.

A total of 1,961 consecutive serum samples from 109 constitutionally healthy children, including the 9 cord blood samples, were studied for HBoV1 IgG and IgM antibodies. All 109 children were seropositive for HBoV1 by age 6 years (Figure 1). Seven children remained seropositive from birth, and 102 children showed seroconversion at the mean age of 2.3 years (median 2.1, range 0.3–6.0) (Table 1, Table 2, Table 3). Ages did not change substantially when calculated according to the mid-point of the seroconversion interval (mean 2.1 years, median 1.9 years, range 0.16–5.7 years). At primary infection of the 102 seroconverters, 53 also showed other markers of HBoV1 infection (Table 2): viremia in 24, IgM antibodies in 28, and low avidity of IgG in 34. In subsequent follow-up, IgG avidity matured in all but 2 children.

**Table 1:** Human bocavirus IgG results from 109 constitutionally healthy children, Finland

Result*	No. (%)	Age, y		
		Mean	Median	Range
Seroconversion	102 (94)	2.30	2.08	0.31–6.00
Secondary response†	38 (35)	4.79	4.77	0.73–9.79
Reconversion	7 (6)	6.29	7.07	1.59–8.15

*Maternal antibodies, i.e., low-level (vanishing) IgG, were detected in 35/88 children from whom serum was taken ≤6 mo. of age. Seven children were IgG positive from birth, and their maternal antibodies were not seen to disappear before induction of their own immunity. For 73, the IgG level remained high; for 26, the IgG level decreased with time; and 10 underwent IgG reversion. †>4-fold increase in, or reconversion of, high avidity IgG (2 children had 2 and 2 other children had 3 secondary immune responses).

**Table 2:** Human bocavirus 1 findings of 102 constitutionally healthy children at seroconversion, Finland*

No. (%) children	Virologic finding	Mean	Median	Range
28 (27)	IgM antibodies, abs	0.69	0.66	0.17–1.54
24 (24)	qPCR positive, copies/mL	1.21 × 10^5^	4.13 × 10^4^	1.26 × 10^0^ to –9.09 × 10^5^
34 (33)	Low IgG avidity, %	8.6	9.4	1.5–14.4

*Abs, absorbance value; qPCR, quantitative PCR in serum.

**Table 3:** Serologic and quantitative PCR results of consecutive serum samples from a representative child, showing all acute HBoV markers, Finland*

Sample no.	Age at sample collection, y	Sampling interval, d	IgG absorbance	IgG interpretation†	IgM absorbance	IgM interpretation‡	IgG avidity, %§	qPCR, copies/mL
1	0.33	120	0.061	Neg	0.015	Neg		
2	0.99	235	0.010	Neg	0.014	Neg		
3	1.25	96	0.029	Neg	0.025	Neg		
4	1.52	96	0.032	Neg	0.023	Neg		
5	1.77	90	0.032	Neg	0.021	Neg		
6	2.02	89	0.017	Neg	0.022	Neg		
7	2.52	180	0.019	Neg	0.028	Neg		
8	2.97	165	0.014	Neg	0.028	Neg	Neg	Neg
**9**	**3.44**	**167**	**1.536**	**Pos**	**0.730**	**Pos**	**2.8**	**7.67 × 10^4^**
10	3.93	178	2.883	Pos	0.023	Neg	60.8	Neg
11	4.46	190	3.412	Pos	0.035	Neg		
12	4.91	163	3.111	Pos	0.022	Neg		
13	5.45	193	3.754	Pos	0.033	Neg		
14	5.96	185	3.228	Pos	0.020	Neg		
15	7.07	398	3.183	Pos	0.030	Neg		
16	7.52	163	3.102	Pos	0.026	Neg		
17	8.07	198	3.450	Pos	0.027	Neg		
18	8.53	165	3.149	Pos	0.016	Neg		
19	9.07	195	3.252	Pos	0.024	Neg	49.2	

*This child experienced an acute HBoV infection at 3 years of age (sample no. 9), evidenced by: IgG conversion, IgM, low IgG avidity and viremia (**boldface)**. HBoV, human bocavirus; qPCR, quantitative PCR in serum; neg, negative; pos, positive; blank cells, not done. †The cutoff absorbances for negative and positive IgG results were 0.154 (mean + 3 SD) and 0.188 (mean + 4 SD), respectively (*13*). ‡The cutoff absorbances for negative and positive IgM results were 0.136 (mean + 3 SD) and 0.167 (mean + 4 SD), respectively (*13*). §The low- and high-avidity cutoff values: 15% and 25%, respectively (*17*).

Only 2 children showed IgM antibodies in 2 consecutive samples, with intervals of 83 and 174 days. The corresponding intervals for the other children were 74–311 days, median 164. Only 4 children showed transient low-level IgM antibody reactivity (mean absorbance 0.335, median 0.285) in 1 serum sample long after seroconversion. One of these 4 IgM-ambiguous samples also was falsely IgM antibody positive for B19 parvovirus, which suggested nonspecificity. None of them contained HBoV1 DNA, but all contained high-avidity HBoV1 IgG antibodies, indicating preexisting immunity.

**Figure 2:**
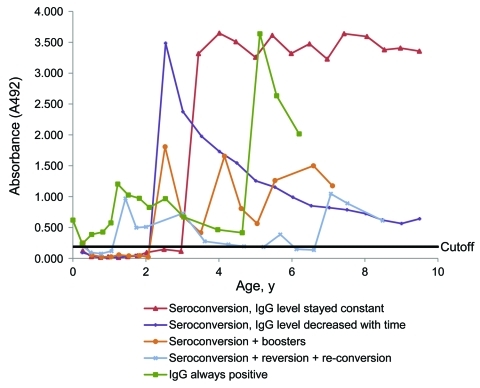
IgG responses in follow-up serum samples from 5 representative children in a study of human bocavirus 1 infection, Finland.

In 73 (67%) of the 109 children, IgG antibody levels after seroconversion remained stable or decreased only slightly during follow-up (Table 1, Table 3; Figure 2). They decreased substantially over time in 26 (24%) children, 10 of whom turned seronegative during follow-up. In all, 38 (35%) children had >1 (44 in total) diagnostic secondary HBoV1 immunoactivations (>4-fold increase in IgG antibody titer in 2 consecutive samples; Figure 1), including 7 reconversions (Figure 2). None of the 44 serum pairs showing secondary responses contained HBoV1 DNA or IgM antibodies (the latter with 1 exception; the nonspecific B19 IgM-reactive serum), and 42 contained IgG of high avidity. In addition, some of the children showed nondiagnostic fluctuations in the IgG antibody level.

All 7 children who lacked a primary seroconversion had been IgG positive from birth (Figure 2). Their umbilical cord samples showed high IgG avidity and lacked IgM and viral DNA. During follow-up, each of these 7 children experienced >1 (2 children had 3) secondary IgG increases within their first 5 years of life.

For 88 children, the first samples were taken during the first 6 months (median 3.7 months of life); 35 (40%) had maternal IgG antibodies (Table 1). One child in this age group showed an HBoV1 primary infection (at 3.7 months): presence of IgM antibodies, IgG conversion, and viremia. An episode of AOM preceded the viremic sample. Another child had a borderline IgM antibody result in her first available sample but exhibited waning (maternal) IgG antibodies. However, umbilical cord blood samples from both of these children were negative for IgM antibodies and DNA, and IgG antibodies were of high avidity, ruling against congenital infection.

### Real-time qPCR

At primary infection, viral DNA was detectable in the serum of 24 of the 102 children with a mean of 1.21 × 10^5^ copies/mL (Table 2). However, none of the serum samples with secondary immune responses was PCR positive. No child had HBoV1 DNA in 2 consecutive samples (postviremic sampling interval 74–207 days, mean 128 days, median 96 days).

### Clinical Correlates

Infection-related symptoms were reported in 90% of the children during the HBoV1 primary infection and in 70% during the subsequent sampling interval (Table 4) (p = 0.0003). When comparing individual symptoms, 2 conditions, URTI (60%) and AOM (47%), occurred significantly more frequently during the HBoV1 primary infection than during the subsequent interval (36% and 31%; p = 0.0002 and p = 0.026). LRTI was also more frequent during the HBoV1 primary infection (5%) than during the next interval (0%) but was too rare for the difference to reach significance; however, when combined with URTI, the difference was highly significant (p<0.0001). The secondary immune responses, including reconversions, showed no association with symptoms (Table 4). The results were similar, regardless of whether the symptoms of HBoV1 infection were compared with those of the previous or the subsequent interval (Table 4). Neither the previous nor the subsequent sampling interval differed significantly in length from the seroconversion interval. The mean differences in length compared with the seroconversion interval were the following: –16.6 days for the previous and +0.5 days for the subsequent interval. 

**Table 4:** Infection-related signs and symptoms during human bocavirus 1 primary seroconversions and secondary responses compared with the previous and subsequent sampling interval, Finland*

Sign or symptom	Primary immune response, n = 101							Secondary immune response, n = 43					
	Interval,† no. (%)	Previous interval‡			Next interval§			Interval,¶ no. (%)	Previous interval#			Next interval**	
		No. (%)	p value		No. (%)	p value			No. (%)	p value		No. (%)	p value
URTI	61 (60.4)	34 (33.7)	**0.0002**		36 (35.6)	**0.0002**		24 (55.8)	21 (48.8)	0.53		23 (53.5)	1
LRTI	5 (4.9)	2 (2.0)	0.45		0	–		1 (2.3)	3 (7.0)	0.62		2 (4.6)	1
URTI or LRTI	62 (61.4)	36 (35.6)	**0.0003**		36 (35.6)	**<0.0001**		24 (55.8)	22 (51.2)	0.83		23 (53.5)	1
Fever without RTI	14 (13.9)	9 (8.9)	0.30		10 (9.9)	0.54		7 (16.3)	3 (7.0)	0.22		3 (7.0)	0.34
Acute otitis media	47 (46.5)	33 (32.7)	**0.024**		31 (30.7)	**0.026**		12 (27.9)	8 (18.6)	0.45		5 (11.6)	0.06
Acute tonsillitis	0	1 (1.0)	–		1 (1.0)	–		1 (2.3)	1 (2.3)	1		0	–
Acute conjunctivitis	5 (4.9)	3 (3.0)	0.72		8 (7.9)	0.51		1 (2.3)	5 (11.6)	0.12		2 (4.6)	1
Acute sinusitis	2 (2.0)	0	–		1 (1.0)	1		2 (4.6)	3 (7.0)	1		2 (4.6)	–
Gastroenteritis	23 (22.8)	19 (18.8)	0.62		16 (15.8)	0.30		9 (20.9)	8 (18.6)	1		10 (23.3)	1
Exanthema, fever	8 (7.9)	4 (4.0)	0.34		0	–		0	1 (2.3)	–		0	–
Other	8 (7.9)	3 (3.0)	0.23		6 (5.9)	0.79		4 (9.3)	2 (4.6)	0.62		3 (7.0)	1
Totals	91 (90.1)	74 (73.3)	**0.003**		71 (70.3)	**0.0003**		35 (81.4)	30 (69.8)	0.27		32 (74.4)	0.58

*URTI, upper respiratory tract illness; LRTI, lower respiratory tract illness; RTI, respiratory tract infection; –, could not be calculated. **Boldface** indicates statistical significance by Liddell exact test (<0.05). †Length of intervals, d: mean 155, median 166, range 75–361; mean age 2.3 y, median age 2.1 y. ‡Length of intervals, d: mean 139, median 130, range 73–358. §Length of intervals, d: mean 155, median 169, range 61–615. ¶Length of intervals, d: mean 188, median 176, range 78–537, mean age 4.8 y, median 4.8 y. #Length of intervals, d: mean 181, median 182, range 61–415. **Length of intervals, d: mean 183, median 176, range 88–408. One child was omitted because of lack of clinical information for 1 interval.

Stability and decline in HBoV1 IgG absorbance level in long-term follow-up were not associated either with diabetes-related autoantibody positivity, allergic disease, or symptoms during HBoV1 primary infection. They were also not associated with excess susceptibility to infections in general.

## Discussion

By comprehensive serologic and molecular testing and follow-up, we observed that by 6 years of age, all children were infected with HBoV1. Reports state that HBoV1 infects predominantly children, and at a young age (*2**,**3*), but almost all studies have been symptom and PCR based and cross-sectional, whereas our study was serum based and longitudinal and spanned the entire period from infancy through 13 years of age.

We determined the seroepidemiology and clinical correlates of HBoV1 infections and the kinetics of HBoV1 infection markers in sequential serum samples from constitutionally healthy children. We showed that HBoV1 primary infections, but not secondary immunoactivations, were significantly associated with respiratory illness and with AOM.

A definitive IgG seroconversion was evident in 102 of the 109 children, half of whom showed further markers of HBoV1 primary infection: viremia, IgM antibodies, or low avidity of IgG. The frequency of viremia or IgM antibody positivity in children with HBoV1 primary infection was considerably lower here than in our earlier study in which 45 of 48 wheezing children with serologically verified HBoV1 primary infection were viremic, and all but 1 who seroconverted had IgM antibodies (*13*). Unlike our current population-based study, our earlier study comprised symptomatic children who gave samples at short intervals during their acute disease. Our PCR results are concordant with the earlier results that showed the brevity of HBoV1 viremia. IgM antibodies persisted slightly longer than did the viremia and were detectable in one fourth of the first IgG-positive serum samples. For human parvovirus B19 (B19V), another pathogenic human parvovirus, the kinetics are the reverse, with viremia usually outlasting IgM antibodies and persisting at a low level for months or years (*22*). In longevity of diagnostic findings, the 3 assays for HBoV1 ranked in this order: IgG-avidity EIA, IgM EIA, serum PCR.

Regarding the high HBoV1 IgG antibody seroprevalence, which has exceeded 90% in adults (*13**,**23**–**26*), we recently observed HBoV1 IgG secondary responses in a large proportion of immunocompetent adults (*17*). In the current study, two thirds of children maintained steady IgG antibody levels for years after seroconversion. In one fourth, however, antibody levels declined substantially with time, and in some cases fell below the detection limit. In most of the children with such a reversion, the HBoV1 IgG antibodies later reconverted. Altogether, 38 (35%) children exhibited diagnostic secondary HBoV1 IgG antibody responses: in 2 children 2× and in another 2 children 3×. The secondary immunoactivations were generally of high avidity and lacked IgM antibodies and were always nonviremic. If these events represent HBoV1 secondary infections, they must be local rather than systemic infections, or they produce short-lived or low-titer viremia that escaped PCR detection. That they were nonviremic would agree with the possibility of B cell boosting by related viruses. The most plausible candidates for such closely related immunogens are the recently discovered HBoV species HBoV2–4 (*4**–**6*), or even more intriguingly, some currently unknown viruses. Past-immunity IgG antibodies against HBoV1–4 cross-react; however, both IgM and IgG antibodies of the acute phase are HBoV1 specific (*27*).

Seven children remained HBoV IgG antibody positive from birth through follow-up, without any observable seroconversion or other acute HBoV-infection markers. The absence of HBoV IgM antibodies and DNA from their umbilical cord blood samples argues against congenital infection. This is in line with a recently noted absence of HBoV from amniotic fluid or fetal tissues (*28**,**29*). The lengths of the sampling intervals may have concealed the disappearance of maternal antibodies and the appearance of markers for HBoV acute infection in the infant. Alternatively, instead of a full replicative infection, the virus could induce a vaccination-like immunity because of preexisting maternal antibodies. Within the first months of life, maternal HBoV IgG antibodies have been observable at prevalences of 26%–78%, depending on the child’s age (*13**,**23**,**24**,**30*), similar to the 40% at a mean age of 4 months in our study. Because only 1 child had detectable HBoV primary infection during the first 6 months of life, the maternal antibodies as a rule seem to be protective—or the infants were less exposed.

The first HBoV prevalence studies showed low detection frequencies of HBoV DNA in the respiratory tracts of asymptomatic children and a high co-infection rate (*31**–**33*). However, subsequent studies indicated a prolonged and frequent presence of HBoV DNA also in asymptomatic children (*7**–**11*). Because of these features and the frequent co-detection of other viruses, the role of HBoV as a respiratory pathogen has been questioned. In a recent prospective study of children in day care, neither HBoV DNA presence nor its load in nasal swabs was associated with the presence or severity of respiratory illness (*11*). On the other hand, low-load HBoV PCR positivity in the upper respiratory tract does not reliably indicate acute HBoV infection, and therefore an accurate diagnosis of acute HBoV infection requires a serum specimen (*12**–**16*).

We collected consecutive serum samples from constitutionally healthy children and recorded the appearance of HBoV antibodies. The parents were interviewed during each sample-collection visit about the child's symptoms within that interval. We compared symptoms during the seroconversion interval with those during the subsequent interval for each child, and, taking into account the slight age difference and variations in occurrences of other respiratory viruses, also to symptoms during the previous sampling interval. With both approaches, HBoV primary infection was unambiguously associated with URTI and with combined URTI and LRTI, strongly suggesting that HBoV does cause respiratory illness. Furthermore, our result linking AOM with HBoV primary infection, in concert with 2 previous studies’ detection of HBoV DNA in middle ear fluid (*34**,**35*), indicates a close association for HBoV also with AOM and middle ear effusion. That these children were genetically susceptible to type 1 diabetes (*18*) most likely does not affect this interpretation of our data.

Even though the strength of our study is the close monitoring of children over a long period (1996–2006), the sampling intervals were too long for any detailed analysis of the seasonal distribution of the infections. However, even distribution throughout the year was evident, which reduced the possibility that HBoV1 infections would accumulate within peak season(s) of other particularly pathogenic viruses, which, at least in theory, could have resulted in a false disease association. Although we did not screen for other respiratory pathogens, the scheduled sampling according to the ages of the children instead of seasons further reduced the possibility of seasonal bias.

In our longitudinal study, all children acquired HBoV infection by 6 years of age. Although most of them subsequently maintained stable HBoV IgG antibody levels, in one fourth, levels substantially declined, and some children lost their antibodies completely, with subsequent reconversion. Secondary infections or anamnestic immune responses commonly occurred. Among the children with HBoV primary infection, >60% had respiratory symptoms. Whereas HBoV primary infections were strongly associated with respiratory illness, the secondary immunoactivations were not. Our results indicate that HBoV1 is a true and common respiratory pathogen.
