# Correction: Vol. 18, No. 2

**DOI:** 10.3201/eid2209.C12209

**Published:** 2016-09

**Authors:** Mira Meriluoto, Lea Hedman, Laura Tanner, Ville Simell, Marjaana Mäkinen, Satu Simell, Juha Mykkänen, Jan Korpelainen, Olli Ruuskanen, Jorma Ilonen, Mikael Knip, Olli Simell, Klaus Hedman, Maria Söderlund-Venermo

**Affiliations:** University of Helsinki, Helsinki, Finland (M. Meriluoto, L. Hedman, M. Knip, K. Hedman, M. Söderlund-Venermo);; Helsinki University Central Hospital, Helsinki (L. Hedman, M. Knip, K. Hedman);; University of Turku, Turku, Finland (L. Tanner, V. Simell, M. Mäkinen, S. Simell, J. Mykkänen, J. Korpelainen, O. Ruuskanen, J. Ilonen, O. Simell);; University of Eastern Finland, Kuopio, Finland (J. Ilonen);; Tampere University Hospital, Tampere, Finland (M. Knip);; Folkhälsan Research Center, Helsinki (M. Knip)

**Keywords:** correction, human bocavirus 1, bocavirus, viruses, infection, respiratory disease, respiratory infections, children, follow-up study, seroconversion, enzyme immunoassay, EIA, IgG, Finland

**To the Editor:** In our earlier assessment of the etiologic role of human bocavirus 1 (HBoV1) among 109 constitutionally healthy children, seroconversion measured by a standard IgG enzyme immunoassay (EIA) was the prime marker of acute infection, and signs and symptoms during sampling intervals were interpreted from hand-written questionnaires and study nurse notes (*1*). In that study, we found that acute HBoV1 infection was associated with upper respiratory tract illness (URTI) and acute otitis media. However, after discovery of 3 new human bocaviruses (HBoV2–4), we used a competitive second-generation IgG EIA to differentiate between type-specific and cross-reacting IgG responses and reassessed the etiologic associations of each virus in the same pediatric cohort. We found only a weak correlation of HBoV1 infection with acute otitis media and no correlation with URTI (*2*).

There are 2 reasons for this discrepancy. First, the competitive second-generation EIA generated a different set of HBoV1-infected children. A total of 27 children initially interpreted as having HBoV1 seroconversions had, on reassessment, HBoV2 or HBoV3 seroconversions (*2*). Second, after improved reevaluation of clinical symptoms of the entire replenished cohort by 3 of the co-authors, we found that URTI symptoms of 38 children were classified differently. Despite tight sampling, intervals of 3 and 6 months might still have been too long for clinical assessment for young children with numerous respiratory infections per year. The authors regret any inconvenience to readers.
